# *Schistosoma japonicum* peptide SJMHE1 suppresses airway inflammation of allergic asthma in mice

**DOI:** 10.1111/jcmm.14661

**Published:** 2019-09-09

**Authors:** Wenzhe Zhang, Li Li, Yu Zheng, Fei Xue, Mengzhu Yu, Yongbin Ma, Liyang Dong, Zirui Shan, Dingqi Feng, Ting Wang, Xuefeng Wang

**Affiliations:** ^1^ Department of Central Laboratory The Affiliated Hospital of Jiangsu University Zhenjiang China; ^2^ Department of Pediatrics The Affiliated Hospital of Jiangsu University Zhenjiang China; ^3^ Department of Neurology Laboratory Jintan Hospital Jiangsu University Zhenjiang China; ^4^ Department of Nuclear Medicine and Institute of Oncology The Affiliated Hospital of Jiangsu University Zhenjiang China

**Keywords:** airway inflammation, allergic asthma, *Schistosoma japonicum* peptide, SJMHE1, suppress

## Abstract

Helminths and their products can shape immune responses by modulating immune cells, which are dysfunctional in inflammatory diseases such as asthma. We previously identified SJMHE1, a small molecule peptide from the HSP60 protein of *Schistosoma japonicum*. SJMHE1 can inhibit delayed‐type hypersensitivity and collagen‐induced arthritis in mice. In the present study, we evaluated this peptide's potential intervention effect and mechanism on ovalbumin‐induced asthma in mice. SJMHE1 treatment suppressed airway inflammation in allergic mice, decreased the infiltrating inflammatory cells in the lungs and bronchoalveolar lavage fluid, modulated the production of pro‐inflammatory and anti‐inflammatory cytokines in the splenocytes and lungs of allergic mice, reduced the percentage of Th2 cells and increased the proportion of Th1 and regulatory T cells (Tregs). At the same time, Foxp3 and T‐bet expression increased, and GATA3 and RORγt decreased in the lungs of allergic mice. We proved that SJMHE1 can interrupt the development of asthma by diminishing airway inflammation in mice. The down‐regulation of Th2 response and the up‐regulation of Th1 and Tregs response may contribute to the protection induced by SJMHE1 in allergic mice. SJMHE1 can serve as a novel therapy for asthma and other allergic or inflammatory diseases.

## 1 INTRODUCTION

Asthma is a chronic inflammatory airway disease. A variety of immune cells and inflammatory mediators (such as eosinophils and Th2 and Th17 cells) and many inflammatory cytokines and chemokines are involved in the airway inflammation of asthma.^1^ Although the pathogenesis of asthma remains unclear, CD4 T cells, especially the imbalance of Th1/Th2 and Th17/regulatory T cells (Tregs), affect the inflammatory responses in asthma.^2^, ^3^ Although corticosteroids can effectively treat the disease, many patients do not respond to the management and suffer from severe long‐term side effects.^4^, ^5^, ^6^ Thus, new therapeutic targets on asthma should be urgently developed.

Research has identified the protective effects of some helminth infections against allergic diseases such as asthma.^7^ Although helminth infections and allergic diseases have similar immune responses, such as high levels of Th2 cytokines, IgE and eosinophilia, the morbidity of asthma in the epidemic areas of parasitic diseases is low.^8^ Helminth infections, such as those caused by *Heligmosomoides polygyrus*, *Schistosoma mansoni* and *Litomosoides sigmodontis*, can modulate airway inflammation in mice.^9^, ^10^, ^11^ Furthermore, the prevalence and severity of asthma in individuals present a decrease in the epidemic area of schistosomiasis.^10^ Schistosoma infection can suppress the development of allergen‐induced airway inflammation in asthmatic mice.^11^, ^12^ Helminth infection and helminth‐derived components can achieve the same effect. In addition to the therapeutic potential of helminths and their products, the immunomodulation induced by helminths may contribute to the identification of the key regulators of pathogenesis in diseases. Schistosoma antigens modulate airway inflammation via IL‐10 and/or CD4^+^CD25^+^Foxp3^+^Tregs in mice^11^, ^13^ and change cytokine secretion and the activation of lymphocytes from asthmatic patients.^14^ However, infectious or whole proteins may elicit side effects to patients. Therefore, seeking small molecules from helminths, such as peptides, for use as immunomodulatory drugs, can be a safe choice for preventing and treating asthma.

In our previous study, we showed that SJMHE1, which is an HSP60‐derived peptide from *Schistosoma japonicum*, could increase CD4^+^CD25^+^ Tregs in vitro and in vivo.^15^ SJMHE1‐ or SJMHE1‐elicited CD4^+^CD25^+^ T cells suppressed delayed‐type hypersensitivity (DTH) in mice.^15^, ^16^ Moreover, SJMHE1 alleviated the inflammation of collagen‐induced arthritis (CIA) in mice.^17^

In the present study, we explored the effects of SJMHE1 on experimental asthma in mice. SJMHE1 treatment significantly decreased the infiltrating inflammatory cells in the airways and regulated the cytokine responses in the splenocytes and lungs of asthmatic mice. SJMHE1 treatment also decreased the population of Th2 cells, along with an increase in Th1 and CD4^+^CD25^+^Foxp3^+^ Tregs, which may protect asthmatic mice from airway inflammation. These results highlight the potential efficacy of SJMHE1 for treating a range of human inflammatory diseases.

## 2 MATERIALS AND METHODS

### 2.1 Mice

Six‐ to eight‐week‐old male BALB/c mice were purchased from the Comparative Medicine Centre of Yangzhou University (Yangzhou, China) and bred under specific pathogen‐free conditions in the Animal Care Facility at Jiangsu University. Animal experiments were performed according to the Guide for the Care and Use of Laboratory Animals and approved by the Animal Research Ethics Committee of Jiangsu University (Permit Number: JSU 16‐127).

### 2.2 Peptides

SJMHE1 peptide from SjHSP60 437‐460 (VPGGGTALLRCIPVLDTLSTKNED) was synthesized and purified from Top‐peptide (Shanghai, China); possible LPS contamination was avoided by using polymyxin B‐agarose as described previously.^17^

### 2.3 SJMHE1 treatment and induction of experimental asthma

Experimental asthma was induced according to a previous study.^18^ Apart from the PBS group, the mice were immunized by three intraperitoneal injections of alum‐precipitated antigen (0.2 mL) containing 50 µg of OVA (fraction V; Sigma, Poole, UK) and 2 mg of 10% aluminium hydroxide gel in PBS on days 0, 7 and 14. They were treated with emulsified SJMHE1 (10 µg) or PBS with incomplete Freund's adjuvant (Sigma, Poole, UK) on days 0, 14 and 28. From days 21 to 27, the mice were challenged with aerosolized OVA (2%) or PBS for 30 minutes using a ME‐U12 ultrasonic nebulizer (Omron, Tokyo, Japan). They were also grouped on the basis of treatment as follows: PBS group, PBS immunized and challenged; OVA group, OVA immunized and challenged; OVA/PBS group, PBS treated and OVA immunized and challenged; OVA/SJMHE1 group, SJMHE1 treated and OVA immunized and challenged. Finally, on day 35, the mice were anaesthetized and killed to evaluate lung and airway inflammation.

### 2.4 Analysis of cells in bronchoalveolar lavage fluid (BALF)

The left bronchial tubes of the mice were ligated, and the right lungs were washed twice using sterile PBS (0.5 mL) to collect the BALF. Then, the BALF was centrifuged. Cell pellets were resuspended in 1 mL PBS, and the total quantity of inflammatory cells was counted using a haemocytometer. For eosinophils count, a smear of the cell pellet of the BALF was prepared and examined by Wright and Giemsa staining.

### 2.5 Histopathologic analysis

After BALF collection, the left lungs were fixed in 10% formalin and embedded in paraffin. Haematoxylin and eosin (H&E) staining was employed to stain the lung tissue of mice. The inflammation of the lungs in mice and histological analyses were determined as previously described.^19^

### 2.6 Immunohistochemical staining

Briefly, lung tissue sections were subjected to antigen retrieval and then blocked and incubated with 1:150 dilution of monoclonal anti Foxp3 antibody (eBioscience) and 1:300 dilution of anti‐rat IgG‐horseradish peroxidase thereafter (Servicebio). Images were captured using Olympus BX51 microscope (Olympus). Image‐Pro Plus 6.0 software (Media Cybernetics) was used to quantify the mean densities of Foxp3, which was stained brown in pixels at 200 × magnification.

### 2.7 Serum anti‐OVA‐specific IgE measurement

The quantification of anti‐OVA‐specific IgE in the sera of mice was carried out by enzyme‐linked immunosorbent assay (ELISA). Briefly, the ELISA plates (Costar) were coated with 100 µL/well OVA (100 µg/mL) in pH 9.6 carbonate‐bicarbonate buffer at 4°C overnight and then blocked for 2 hours with 200 µL/well skimmed milk powder (5%). After washing, the diluted sera were added and incubated for 2 hours at 37°C. After washing, 1:250 dilution of goat anti‐mouse IgE (Abcam) was added and incubated for 2 hours at 37°C. After removing the unbound antibodies, HRP‐conjugated rabbit anti‐goat secondary IgG (1:5000, Multisciences) was incubated for 1 hours at 37°C. The colour reaction was developed by adding 100 µL/well of TMB solution (eBioscience) for 15 minutes and then stopped with 50 µL/well of 2 M sulphuric acid. Finally, it was read at 450 nm in an ELISA reader (Bio‐Rad).

### 2.8 Flow cytometry analysis

Splenocytes from mice were suspended in the presence of PMA/ionomycin mixture (Phorbol 12‐myristate13‐acetate, Multisciences) and brefeldin/monensin mixture (Multisciences) for 5 hours to analyse the Th1, Th2 and Th17 cells. Then, the cells were collected and stained with PerCP anti‐CD3 mAbs (eBioscience) and FITC anti‐CD4 mAbs (eBioscience). After removing the unbound antibodies, the cells were fixed and permeabilized with Cytofix/Cytoperm (BD Biosciences). Then, they were stained with APC mouse anti‐IFN‐γ (eBioscience), phycoerythrin (PE) mouse anti‐IL‐4 (BioLegend) and PE mouse anti‐17A (BioLegend) following the manufacturer's instructions. To determine Tregs, we used the Mouse Regulatory T Cell Staining Kit (eBioscience) for analysis as previously described.^15^ The samples were analysed using BD FACSCanto flow cytometer (BD Biosciences) and Flowjo Software (Tree Star).

### 2.9 RNA extraction and quantitative RT‐PCR (qRT‐PCR)

The extraction of RNA and the reverse transcription from splenocytes and lungs of mice were performed using Prime Script 1st Strand cDNA Synthesis Kit (Takara). A quantitative analysis of the relative mRNA expression of cytokines was performed in the spleen cells and lung tissue by qRT‐PCR using All‐in‐one™ Mix (Genecopoeia). All‐in‐one™ qPCR primer sets for IFN‐γ (cat. no. MQP027401), IL‐4 (cat. no.MQP032451), IL‐5 (cat. no. MQP029462), IL‐17 (cat.no. MQP029457), IL‐10 (cat. no. MQP029453), TGF‐β (cat. no. MQP030343), IL‐35 (cat. no. MQP027412) and Foxp3 (cat. no. MQP067272) (Genecopoeia) were used, and mouse GAPDH (cat. no. MQP027158) was used as an endogenous control for sample normalization. The parameters for PCR amplification were 95°C for 10 minutes, followed by 40 cycles of 95°C for 10 seconds, 60°C for 20 seconds and 72°C for 15 seconds. The relative mRNA expression was calculated with the comparative △Ct method using the formula 2^−△△Ct.^

### 2.10 Western blot analysis

The proteins of the lung tissues in mice were extracted for Western blot analysis as described previously.^20^ Rat monoclonal antibodies of anti‐T‐bet (eBioscience) (1:250 dilution), anti‐GATA‐3 (Cell Signaling Technology) (1:1000 dilution), anti‐RORγ(t) (eBioscience) (1:100 dilution) and GAPDH (Cell Signaling Technology) (1:1000 dilution) were used as the primary antibodies. HRP‐conjugated anti‐rat IgG (Beyotime Biotechnology) was also utilized. ECL chemiluminescence kit was used for chemiluminescent detection followed by image analysis.

### 2.11 Statistical analyses

Statistical analyses were performed with GraphPad Prism 5.01 (GraphPad Software, 2007). Data were expressed as mean ± standard error of the mean. The groups were compared using one way ANOVA with Tukey Kramer post hoc tests, in which a *P*‐value of less than 0.05 was considered as statistically significant.

## 3 RESULTS

### 3.1 SJMHE1 treatment suppresses OVA‐induced inflammation in allergic mice

**Figure 1:**
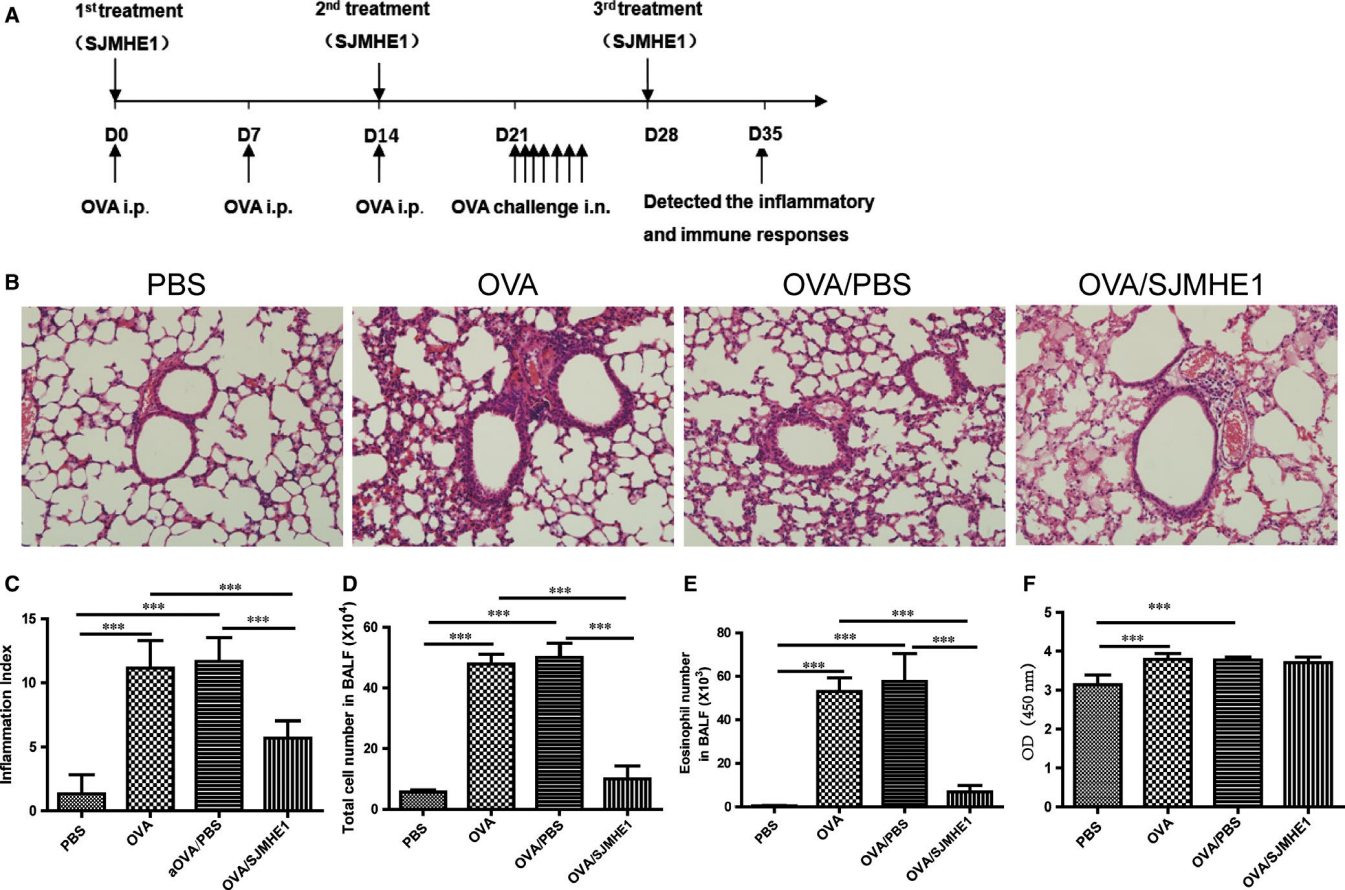
SJMHE1 treatment inhibits the development of airway inflammation in allergic mice. A, Experimental scheme. BALB/c mice were sensitized with OVA on days 0, 7 and 14 and challenged with OVA daily from days 21 to 27. Mice were injected with SJMHE1 emulsified in IFA on days 0, 14 and day 28. The mice were killed on day 35. B, Histological analysis of lung section from mice by H&E staining. 20× magnification. Images are representative of four independent experiments (n = 6 mice per group). C, Inflammatory process was scored following Underwood's standards, with 15 indicating the most severe pathological changes for each mouse. D, Total cells in BALF. E, Eosinophil number in BALF. The quantity of eosinophil in BALF cells was determined following Wright and Giemsa staining. F, OVA‐specific IgE antibody levels in sera of mice. Results are presented as mean ± SEM (n = 12) from two‐independent experiments. ^***^ *P* < .001

Mice were sensitized and challenged by OVA to examine the effects of SJMHE1 on asthma. The treatment regimen is illustrated in Figure 1A. As shown in Figure 1B, asthmatic mice displayed intense inflammatory cell infiltration in the peribronchial and perivascular regions of the lungs. Meanwhile, SJMHE1 treatment reduced the cellular infiltration of the lungs. The inflammation index was determined using Underwood's standards as described previously.^19^ On the basis of inflammation indexes, the OVA and OVA/PBS groups showed more perivascular eosinophilia, epithelial damage and oedema compared with the PBS control group (Figure 1B and 1C). However, compared with those in the OVA or OVA/PBS group, the inflammation scores in SJMHE1‐treated mice were significantly reduced (Figure 1B and 1C). Furthermore, SJMHE1 treatment significantly decreased the infiltrating inflammatory cells induced by OVA sensitization and challenge because the OVA or OVA/PBS group had significantly higher cell and eosinophilia numbers in the BALF than in the OVA/SJMHE1 group (Figure 1D and 1E). OVA‐specific IgE was measured by ELISA. As shown in Figure 1F, the OVA and OVA/PBS groups had significantly higher IgE level than the PBS control group. However, the SJMHE1 treatment group showed no reduction in IgE level relative to the OVA or OVA/PBS group. These results indicate that SJMHE1 treatment can suppress airway inflammation in allergic mice.

### 3.2 SJMHE1 treatment modulates cytokine expression in the splenocytes and lungs of allergic mice

**Figure 2:**
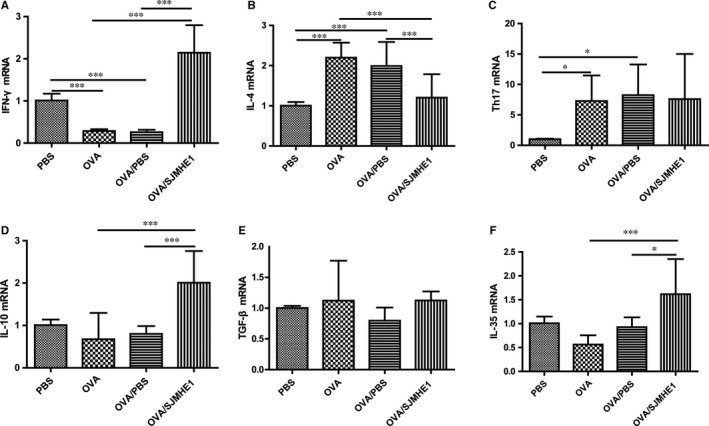
SJMHE1 treatment modulates the expression of cytokines from splenocytes of allergic mice. On day 35, the mice were killed, and the splenocytes from each mouse were tested for mRNA expression of IFN‐γ (A), IL‐4 (B), IL‐17 (C), IL‐10 (D), TGF‐β (E) and IL‐35 (F) by qRT‐PCR. Results are presented as mean ± SEM of 12 mice from two independent experiments performed in triplicate wells. ^*^ *P* < .05, ^***^ *P* < .001

**Figure 3:**
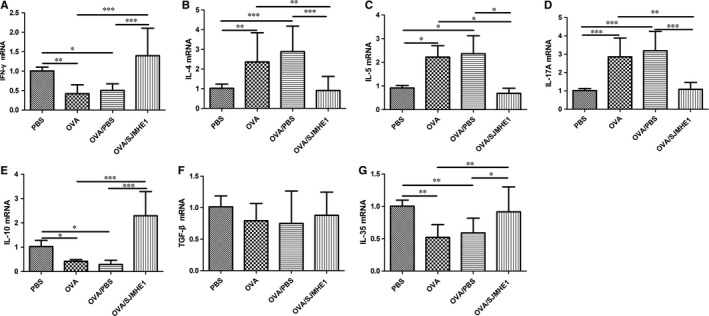
SJMHE1 treatment modulates the expression of cytokines from lungs of allergic mice. On day 35, the mice were killed, and the lungs from each mouse were tested for mRNA expression of IFN‐γ (A), IL‐4 (B), IL‐5 (C), IL‐17 (D), IL‐10 (E), TGF‐β (F) and IL‐35 (G) by qRT‐PCR. Results are presented as mean ± SEM of 12 mice from two independent experiments performed in triplicate wells. ^*^ *P* < .05, ^**^ *P* < .01, ^***^ *P* < .001

The elevation of Th2 and Th17 cytokines, such as IL‐4 and IL‐17, in asthmatic patients may affect the pathogenesis of asthma.^2^ Parasitic infections and their products can protect against inflammatory diseases by inducing immunomodulatory cytokines, such as IL‐10, TGF‐β and IL‐35.^21^ To investigate the effects of cytokines on SJMHE1 treatment, we tested the expression of cytokines in the splenocytes and lungs of mice. As shown in Figure 2, the OVA and OVA/PBS groups presented more IL‐4 and IL‐17 mRNA and lesser IFN‐γ mRNA in the splenocytes than the PBS control group (Figure 2A‐C). However, a decrease in IL‐4 mRNA and an increase in IFN‐γ, IL‐10 and IL‐35 mRNA were detected in the splenocytes of OVA/SJMHE1‐treated mice (Figure 2). Meanwhile, higher levels of IL‐4, IL‐5, and IL‐17 mRNA and lower levels of IFN‐γ, IL‐10 and IL‐35 mRNA were observed in the lungs of mice from the OVA and OVA/PBS groups than from the PBS control group (Figure 3). However, the OVA/SJMHE1‐treated mice displayed an increase in IFN‐γ, IL‐10, and IL‐35 mRNA and a decrease in IL‐4, IL‐5 and IL‐17 mRNA in the lungs relative to the OVA‐ and OVA/PBS‐treated mice. In line with the gene expression, analysis of cytokine release confirmed that IL‐5 expression was increased in the splenocytes of OVA‐treated mice. SJMHE1 treatment induced a decrease of IL‐5 expression level in the splenocytes of OVA/SJMHE1‐treated mice. Compared with the PBS group, the IFN‐γ level was slightly reduced in OVA‐ and OVA/PBS‐treated mice, whereas SJMHE1 treatment induced more IFN‐γ production compared with the OVA/PBS group (Figure S1). Similarly, more IL‐4 and IL‐17 expression, and lesser IL‐10 expression were observed in the lungs of OVA‐, and OVA/PBS‐treated mice than in PBS‐treated mice as shown by the results of Western blot analysis and immunohistochemical staining, respectively (Figure S2 and Figure S3). However, SJMHE1 treatment induced a decrease of IL‐4 and IL‐17 expression, and an increase of IL‐10 expression. No difference was observed in TGF‐β expression in lungs of PBS‐, OVA‐, OVA/PBS‐ and OVA/SJMHE1‐treated mice (Figure S3). These results show that SJMHE1 treatment can suppress the production of pro‐inflammatory cytokines and induce the expression of anti‐inflammatory cytokines in the splenocytes and lungs of allergic mice.

### 3.3 SJMHE1 treatment modulates the proportion of Th1/Th2/Th17/Treg subsets in splenocytes of allergic mice

**Figure 4:**
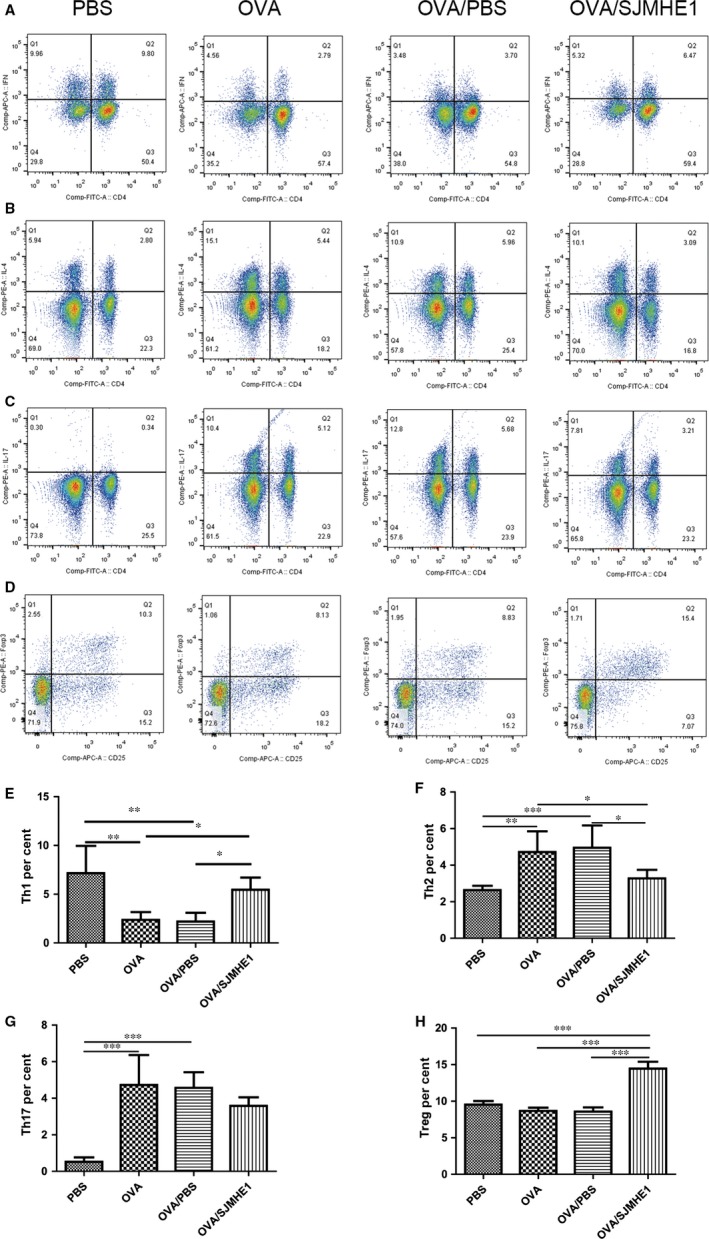
SJMHE1 treatment modulates Th1/Th2/Th17/Treg response in splenocytes of allergic mice. On day 35, the mice were killed, and the splenocytes from each mouse were tested for Th1/Th2/Th17/Treg subsets by flow cytometry. A, CD4^+^IFN‐γ^+^ Th1 cells, B, CD4^+^IL4^+^ Th2 cells, C, CD4^+^IL17^+^ Th17 cells and D, CD4^+^CD25^+^Foxp3^+^ Tregs in each group are shown. Data are representative of the experiments. E, The percentage of CD4^+^IFN‐γ^+^ Th1 cells, F, the percentage of CD4^+^IL4^+^ Th2 cells, G, the percentage of CD4^+^IL17^+^ Th17 cells and H, the percentage of CD4^+^CD25^+^Foxp3^+^ Tregs in each group are shown. Results are presented as mean ± SEM of 12 mice from two independent experiments. ^*^ *P* < .05, ^**^ *P* < .01, ^***^ *P* < .001

An imbalance of Th1/Th2 and Th17/Treg has been observed in allergic patients.^3^ Furthermore, our previous study demonstrated that SJMHE1 treatment can induce CD4^+^CD25^+^Tregs to suppress DTH responses^16^ and alleviate CIA in mice.^17^ In the present study, whether SJMHE1 suppresses airway inflammation and modulates cytokine production in allergic mice by regulating the Th subsets during treatment was examined. Proportions of Th1/Th2/Th17/Tregs in splenocytes from mice in the PBS, OVA, OVA/PBS and OVA/SJMHE1 treatment groups were measured. As shown in Figure 4 (Figure S4 for gating strategy), compared with the PBS control mice, the OVA‐ or OVA/PBS‐treated mice presented increased proportions of CD4^+^IL4^+^ Th2 and CD4^+^IL17^+^ Th17 cells and decreased proportions of CD4^+^IFN‐γ^+^ Th1 cells. However, the proportion of CD4^+^IFN‐γ^+^ Th1 and CD4^+^CD25^+^Foxp3^+^ Treg cells significantly increased, that of CD4^+^IL4^+^ Th2 cells significantly decreased, and that of CD4^+^IL17^+^ Th17 cells slightly decreased without statistical significance in the OVA/SJMHE1 group relative to the OVA or OVA/PBS group. These results indicate that SJMHE1 treatment up‐regulates Th1 and Tregs, possibly providing protection against Th2 and Th17 cells in allergic mice.

### 3.4 SJMHE1 treatment increases the expression of Foxp3 in the splenocytes and lungs of allergic mice

**Figure 5:**
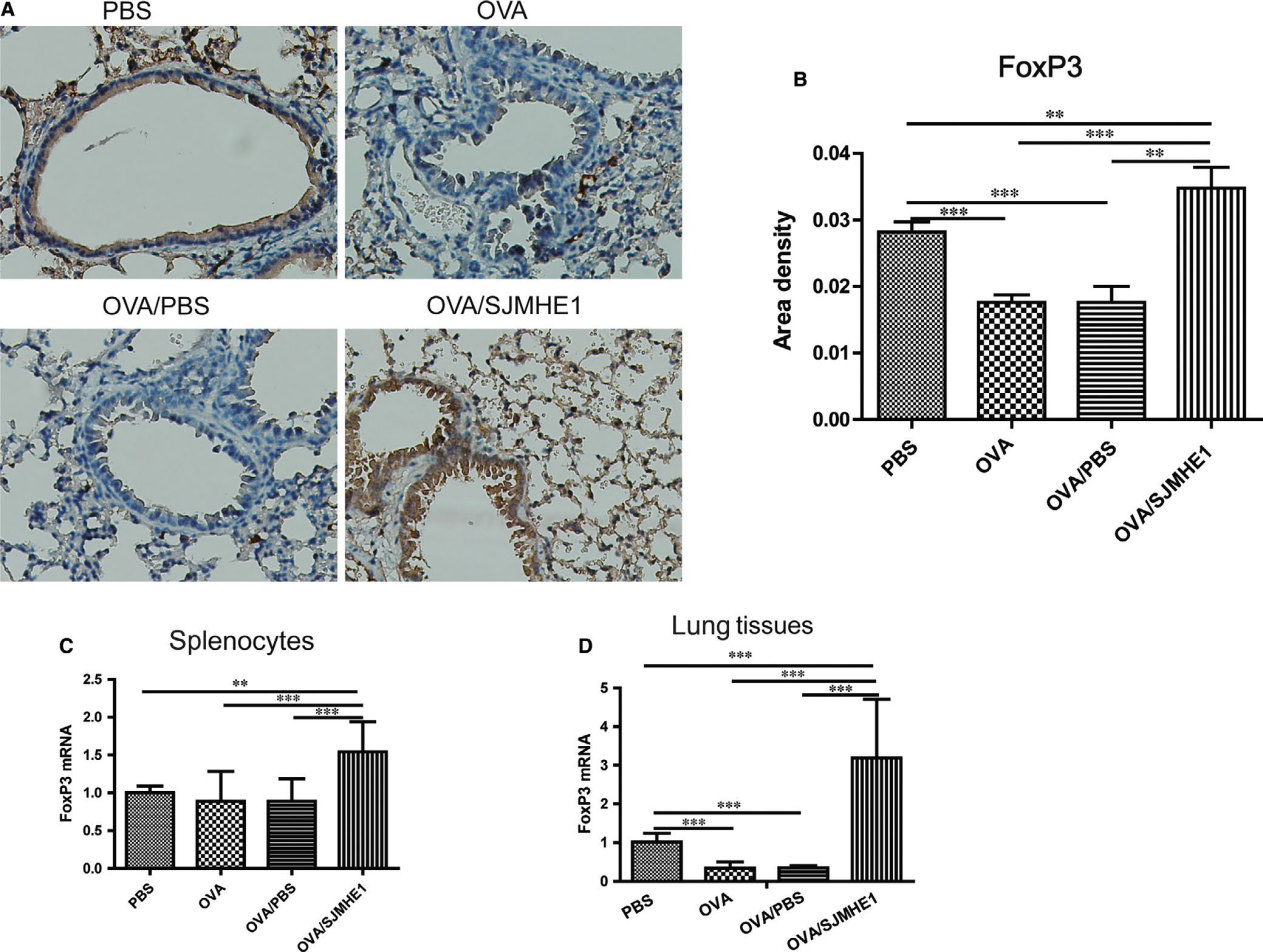
SJMHE1 treatment increases the expression of Foxp3 in splenocytes and lungs of allergic mice. On day 35, the mice were killed, and the splenocytes and lungs from each mouse were tested for Foxp3 expression. A, Foxp3 expression in lungs by IHC staining. Images are representative of two independent experiments (n = 6 mice per group). B, Quantification of Foxp3‐positive areas in each group using Image‐Pro Plus software. Data are presented as mean ± SEM of 12 mice from two independent experiments. C, The expression of Foxp3 mRNA in splenocytes and lungs D, from each group by qRT‐PCR. Data are presented as mean ± SEM of 12 mice from two independent experiments. ^**^ *P* < .01, ^***^ *P* < .001

Foxp3 is the lineage‐specific transcription factor of CD4^+^CD25^+^Tregs. To investigate the generation of Tregs induced by SJMHE1 on the immune system and inflammatory site further, we detected the expression of Foxp3 in the splenocytes and lungs of allergic mice. As shown in Figure 5A and 5B, Foxp3 expression by immunohistochemical staining was decreased in the lungs of the OVA‐ and OVA/PBS‐treated mice relative to the PBS control mice. However, more Foxp3 expression was observed in the lungs of the OVA/SJMHE1‐treated mice than in those of the PBS‐, OVA‐ and OVA/PBS‐treated mice. Specifically, the expression of Foxp3 mRNA decreased in the lungs of the OVA‐ or OVA/PBS‐treated mice relative to the PBS control group. However, a higher level of Foxp3 mRNA was observed in the splenocytes and lungs of the mice from the OVA/SJMHE1 group than in those of the mice from the OVA and OVA/PBS groups (Figure 5C and 5D). These results indicate that high expressions of Foxp3 induced by SJMHE1 may offer protection against inflammatory responses in allergic mice.

### 3.5 SJMHE1 treatment up‐regulates the expression of T‐bet and down‐regulates the expression of GATA3 and RORγt in lungs of allergic mice

**Figure 6:**
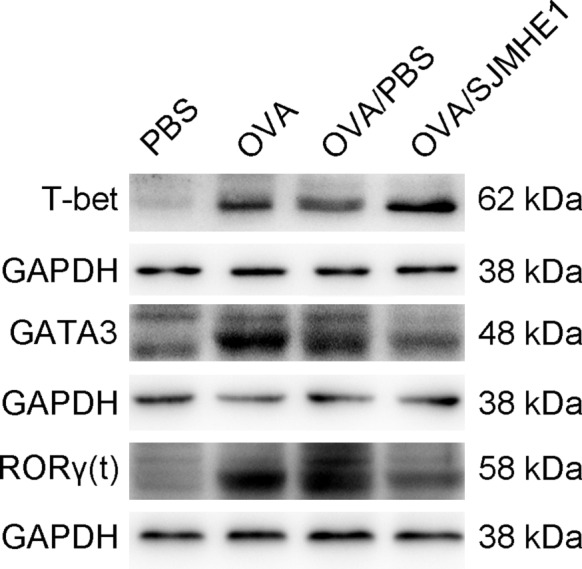
SJMHE1 treatment up‐regulates the expression of T‐bet and down‐regulates the expression of GATA3 and RORγt in lungs of allergic mice. On day 35, the mice were killed, and the lungs from each mouse were tested for T‐bet, GATA3 and RORγt expression by Western blotting analysis. A representative blot from each group is shown

T‐bet, GATA3 and RORγt are the dominant transcription factors of Th1, Th2 and Th17 cells, respectively. To determine whether SJMHE1 treatment affects the expression of these transcription factors at inflammatory sites, we detected their expression in lungs of mice by Western blot analysis. As shown in Figure 6, GATA3 and RORγt were substantially expressed in OVA‐ and OVA/PBS‐treated mice than in the PBS control group. However, SJMHE1 treatment down‐regulated the expression of GATA3 and RORγt and up‐regulated the that of T‐bet; therefore, combined with the increase of Foxp3 expression in Figure 5, SJMHE1 may inhibit the transcription and differentiation of pro‐inflammatory Th2 and Th17 cells by promoting the development of Th1 and Treg cells at the inflammatory sites of allergic mice.

## 4 DISCUSSION

Parasite‐derived molecules regulate the host immune responses with various mechanisms to ensure their survival while inhibiting excessive inflammatory responses, such as suppressing the development of allergic diseases, in their host. Parasite‐derived products may be more effective and safer to humans than other chemical drugs because parasites can parasitize their host for a long time by regulating the immune response of the host.^22^ To date, many commercial companies and private entities have produced and marketed helminths to treat inflammatory diseases.^23^

In the present study, we demonstrated that *S japonicum* peptide SJMHE1 can significantly suppress OVA‐induced airway inflammation and reduce inflammatory cells, including eosinophil infiltration into the airways. However, the reduction of airway inflammation induced by SJMHE1 is not associated with reduction in IgE levels because SJMHE1 treatment did not alter IgE levels in the OVA, OVA/PBS and OVA/SJMHE1 groups. Helminth infection is known to induce both polyclonal‐ and antigen‐specific IgE production and elicits an IgE‐associated type 2 immune response.^24^ As a *S japonicum*‐derived peptide, SJMHE1 may induce IgE response. These results are consistent with other parasite studies demonstrating that excretory/secretory (ES) and somatic products of *Marshallagia marshalli* reduce inflammatory cell infiltration and suppress OVA‐induced allergic asthma but fail to decrease IgE levels.^25^ Similarly, Kitagaki and colleagues reported that *Heligosomoides polygyrus* infection protects mice against asthma but increases OVA‐specific IgE.^26^ However, many investigations have shown that parasite infections or their products decrease the level of OVA‐specific IgE in murine models of asthma.^13^, ^27^, ^28^, ^29^ These differences may be related to the various parasites or their molecules, intervention methods or disease microenvironments resulting from induction methods.

The immune pathogenesis of asthma is complex. In response to allergens, pollutants and microbes, bronchial epithelial cells produce inflammatory mediators that trigger innate immune cells to release a broad array of cytokines. These cytokines then elicit the development of Th2 and Th17 responses, which further secrete pro‐inflammatory cytokines to perpetuate these responses.^2^ Recent studies have demonstrated that these pro‐inflammatory cytokines undermine the structural integrity and impair the epithelial barrier of the respiratory tract.^2^ Meanwhile, helminths can induce Tregs to regulate inflammatory responses by releasing anti‐inflammatory cytokines, such as IL‐10, TGF‐β and IL‐35.^30^ Thus, in the present study, we detected cytokines from the splenocytes and lungs of asthmatic mice. SJMHE1 treatment significantly reduced the IL‐4 mRNA in the splenocytes and suppressed the expression of IL‐4, IL‐5 and IL‐17 mRNA in the lungs of mice relative to the OVA or OVA/PBS group. Nonetheless, helminth infections or their products induce a type 2 immune response, which produces cytokines, such as IL‐4, IL‐5 and IL‐13. These cytokines orchestrate the differentiation of Th2 cells.^30^, ^31^ ES‐62 from *Acanthocheilonema viteae* is a phosphorylcholine‐containing glycoprotein that can induce IFN‐γ to restore Th1/Th2 balance away from Th2 in airway inflammation while suppressing Th1/Th17 responses in CIA.^32^, ^33^ In our work, we demonstrated that SJMHE1 treatment induces an increase in IFN‐γ, IL‐10 and IL‐35 mRNA levels in the splenocytes and lungs of allergic mice. IL‐35, a newly identified inhibitory cytokine released by Tregs, can suppress the airway inflammation induced by allergen‐specific Th2 cells and IL‐17‐dependent response in allergic mice.^34^, ^35^, ^36^ Furthermore, it induces the production of IL‐10 and IFN‐γ.^37^ In addition to IL‐35, an increase in IFN‐γ and IL‐10 has been observed in *Trichuris muris*‐infected mice, and this increase can inhibit the allergic airway inflammation induced by papain.^38^ Although we did not investigate how IFN‐γ, IL‐10 and IL‐35 affect and produce one another by SJMHE1 treatment, we found that the inhibition of IL‐4, IL‐5 and IL‐17 and elevation of IFN‐γ, IL‐10 and IL‐35 may contribute to the protection conferred by SJMHE1 in allergic mice. This study, which up‐regulated the production of IL‐10 and IL‐35, is different from those on suppression induced by SJMHE1 in DTH and CIA mice.^16^, ^17^ We support the notion that helminths or their products regulate Th1/Th2/Th17‐associated inflammation in different models by modulating distinct targets.^39^ It should be noted that cytokines are produced in splenocytes primarily by CD4 T cells because these cells affect inflammatory responses in asthma.^2^ In line with the results in Figure 4, IFN‐γ decreased, but IL‐4 and IL‐17A increased in OVA and OVA/PBS group than in PBS group gating from CD4 T cells as observed by flow cytometry. SJMHE1 treatment increased IFN‐γ but decreased IL‐4 from CD4 T cells. However, cytokine production in lungs from allergic mice is complex; apart from traditional Th2 cells, the lungs also contain epithelium‐activated group 2 innate lymphoid cells (ILC2s).^40^ To clarify the pathogenesis of asthma, the cell source of cytokines in splenocytes and lungs of allergic mice should be determined in the future.

CD4^+^ T cells participate in the pathogenesis of chronic inflammation in asthma. Consistent with cytokine production from splenocytes of mice, we discovered a low level of Th1 cells and a high level of Th2 and Th17 cells in the splenocytes of OVA‐ or OVA/PBS‐treated mice. However, in contrast to studies that found Tregs from asthma patients are dysfunctional and can suppress allergic diseases in humans and animals,^41^, ^42^ we did not observe a decrease in Tregs in the splenocytes of OVA‐ or OVA/PBS‐treated mice relative to the PBS control group. Correspondingly, OVA or OVA/PBS treatment did not elicit a decrease in Foxp3 mRNA expression in splenocytes but did present a significant decline in Foxp3 mRNA and protein in the lungs relative to the PBS control group (Figure 5). Consistent with previous reports on helminth infections or their products being able to induce the production of Tregs,^8^, ^30^ we discovered that the proportion of CD4^+^CD25^+^Foxp3^+^Tregs in the spleen was increased in SJMHE1‐treated mice. Moreover, OVA/SJMHE1‐treated mice showed an increase in Foxp3 mRNA in the splenocytes and an elevation of Foxp3 mRNA and protein in the lungs relative to the OVA or OVA/PBS group (Figure 5). Such outcome suggested a defect of Tregs in local inflammation in allergic mice. Moreover, SJMHE1 can induce Tregs to suppress Th2 and Th17 responses of allergic mice. Although Foxp3 expression is critical for Tregs to maintain their suppressive function by producing inhibitory cytokines, such as IL‐10, TGF‐β and IL‐35,^43^, ^44^ a recent study showed the existence of a Foxp3‐independent mechanism by TGF‐β‐induced peripheral tolerance.^45^ Furthermore, Foxp3 is necessary for IL‐35 but not essential for IL‐10, whereas activated Treg generates IL‐35‐ and IL‐10‐producing subsets to maintain immune tolerance.^46^ Combined with the expression of IL‐10 and IL‐35 mRNA expression by SJMHE1 treatment in allergic mice (Figures 2 and 3), we can infer that the induction of Tregs by SJMHE1 exerts inhibitory functions in allergic mice through IL‐10 and IL‐35.

Consistent with the expression of IFN‐γ, IL‐4, IL‐5 and IL‐17 mRNA, SJMHE1 treatment increased the expression of T‐bet and decreased the expression of GATA3 and RORγt, which are the dominant transcription factors of Th1, Th2 and Th17 cells, respectively. However, compared with the OVA and OVA/PBS groups, T‐bet expression was down‐regulated in the PBS group, which is inconsistent with the increase of IFN‐γ mRNA expression in the PBS group (Figure 3). The protein level of T‐bet might have undergone ubiquitination degradation in the PBS group in a non‐inflammatory environment.^47^ Although T‐bet plays a critical role for IFN‐γ production in Th1 cells, IFN‐γ secretion from other immune cells, such as natural killer cells in T‐bet deficient mice, is necessary and sufficient to protect the host against *Listeria monocytogenes*.^48^ SJMHE1 appears to reset the balance of effector T cells in inflammatory sites by acting upstream of inflammatory cascades. Thus, the inhibition of inflammation using helminths or their products is a consequence of the natural immune response to helminths. This inflammation inhibition induced by helminths does not target a single molecule or pathway; the suppression is the outcome of the complex and multifaceted immune response, the mechanism of which requires further understanding. We support the notion that helminths or their products can interact with a variety of innate and adaptive immune cells to disrupt pathogenic networks elicited by stromal cells in microenvironmental niches.^39^ In addition to such therapeutic potential, these helminth‐derived molecules can be used to validate the key regulators of disease pathogenesis.^49^ Thus, small molecule peptides such as SJMHE1 from helminths are substantially more acceptable and easily regulated as a therapeutic modality than experimental helminth infections or their intact proteins. This class of biologics has appeal for a variety of inflammatory diseases, such as allergy and autoimmune diseases, which are usually elicited by an imbalance between pro‐ and anti‐inflammatory T cell responses and are pandemics in industrialized and developing nations.

In summary, we demonstrated that SJMHE, a peptide from *S japonicum*, can inhibit airway inflammation in OVA‐induced experimental asthma in mice. The up‐regulation of Tregs and the Th1‐skewed responses induced by SJMHE1 in the immune system and inflammatory sites might provide protection against airway inflammation in allergic mice.

## CONFLICT OF INTERESTS

The authors declare that they have no competing interests. The funding agencies played no role in the design or implementation of the study, analysis or interpretation of the data, or the preparation and submission of the manuscript.

## AUTHORS CONTRIBUTIONS

Conceived and designed the experiments: WZZ LL XFW. Performed the experiments: WZZ LL YZ FX MZY. Analysed the data: YBM LYD ZRS. Contributed reagents/materials/ analysis tools: DQF TW. Wrote the paper: WZZ LL XFW. All authors read and approved the final manuscript.

## References

[1] Lemanske RF Jr , Busse WW . Asthma: clinical expression and molecular mechanisms. J Allergy Clin Immunol. 2010;125:S95‐S102. 20176271 10.1016/j.jaci.2009.10.047 PMC2853245

[2] Muehling LM , Lawrence MG , Woodfolk JA . Pathogenic CD4(+) T cells in patients with asthma. J Allergy Clin Immunol. 2017;140:1523‐1540. 28442213 10.1016/j.jaci.2017.02.025 PMC5651193

[3] Shi YH , Shi GC , Wan HY , et al. Coexistence of Th1/Th2 and Th17/Treg imbalances in patients with allergic asthma. Chin Med J. 2011;124:1951‐1956. 22088452

[4] Gilchrist FJ , Ahmad AN , Batchelor HK , Marriott JF , Lenney W . A review of prednisolone prescribing for children with acute asthma in the UK. J Asthma. 2016;53:563‐566. 27043745 10.3109/02770903.2015.1118498

[5] Choby GW , Lee S . Pharmacotherapy for the treatment of asthma: current treatment options and future directions. Int Forum Allergy Rhinol. 2015;5(Suppl 1):S35‐40. 26335835 10.1002/alr.21592

[6] Cooper V , Metcalf L , Versnel J , Upton J , Walker S , Horne R . Patient‐reported side effects, concerns and adherence to corticosteroid treatment for asthma, and comparison with physician estimates of side‐effect prevalence: a UK‐wide, cross‐sectional study. NPJ Prim Care Respir Med. 2015;25:15026. 26158805 10.1038/npjpcrm.2015.26 PMC4497315

[7] Wammes LJ , Mpairwe H , Elliott AM , Yazdanbakhsh M . Helminth therapy or elimination: epidemiological, immunological, and clinical considerations. Lancet Infect Dis. 2014;14:1150‐1162. 24981042 10.1016/S1473-3099(14)70771-6

[8] Wuhao L , Ran C , Xujin H , Zhongdao W , Dekumyoy P , Zhiyue L . Parasites and asthma. Parasitol Res. 2017;116:2373‐2383. 28689246 10.1007/s00436-017-5548-1

[9] Dittrich AM , Erbacher A , Specht S , et al. Helminth infection with Litomosoides sigmodontis induces regulatory T cells and inhibits allergic sensitization, airway inflammation, and hyperreactivity in a murine asthma model. J Immunol. 2008;180:1792‐1799. 18209076 10.4049/jimmunol.180.3.1792

[10] Medeiros M Jr , Figueiredo JP , Almeida MC , et al. Schistosoma mansoni infection is associated with a reduced course of asthma. J Allergy Clin Immunol. 2003;111:947‐951. 12743556 10.1067/mai.2003.1381

[11] Pacifico LG , Marinho FA , Fonseca CT , et al. Schistosoma mansoni antigens modulate experimental allergic asthma in a murine model: a major role for CD4+ CD25+ Foxp3+ T cells independent of interleukin‐10. Infect Immun. 2009;77:98‐107. 18824533 10.1128/IAI.00783-07 PMC2612239

[12] Mo HM , Lei JH , Jiang ZW , et al. Schistosoma japonicum infection modulates the development of allergen‐induced airway inflammation in mice. Parasitol Res. 2008;103:1183‐1189. 18654798 10.1007/s00436-008-1114-1

[13] Marinho FV , Alves CC , de Souza SC , et al. Schistosoma mansoni tegument (Smteg) induces IL‐10 and modulates experimental airway inflammation. PLoS ONE. 2016;11:e0160118. 27454771 10.1371/journal.pone.0160118 PMC4959726

[14] de Almeida T , Fernandes JS , Lopes DM , et al. Schistosoma mansoni antigens alter activation markers and cytokine profile in lymphocytes of patients with asthma. Acta Trop. 2017;166:268‐279. 27931742 10.1016/j.actatropica.2016.12.002

[15] Wang X , Zhou S , Chi Y , et al. CD4+CD25+ Treg induction by an HSP60‐derived peptide SJMHE1 from Schistosoma japonicum is TLR2 dependent. Eur J Immunol. 2009;39:3052‐3065. 19882655 10.1002/eji.200939335

[16] Wang X , Wang J , Liang Y , et al. Schistosoma japonicum HSP60‐derived peptide SJMHE1 suppresses delayed‐type hypersensitivity in a murine model. Parasit Vectors. 2016;9:147. 26971312 10.1186/s13071-016-1434-4 PMC4789290

[17] Wang X , Li L , Wang J , et al. Inhibition of cytokine response to TLR stimulation and alleviation of collagen‐induced arthritis in mice by Schistosoma japonicum peptide SJMHE1. J Cell Mol Med. 2017;21:475‐486. 27677654 10.1111/jcmm.12991 PMC5323857

[18] Du Q , Chen Z , Zhou LF , Zhang Q , Huang M , Yin KS . Inhibitory effects of astragaloside IV on ovalbumin‐induced chronic experimental asthma. Can J Physiol Pharmacol. 2008;86:449‐457. 18641694 10.1139/y08-053

[19] Underwood S , Foster M , Raeburn D , Bottoms S , Karlsson JA . Time‐course of antigen‐induced airway inflammation in the guinea‐pig and its relationship to airway hyperresponsiveness. Eur Respir J. 1995;8:2104‐2113. 8666107 10.1183/109031936.95.08122104

[20] Ma X , Sun Z , Zhai P , et al. Effect of follicular helper T cells on the pathogenesis of asthma. Exp Therapeutic Med. 2017;14:967‐972. 10.3892/etm.2017.4627 PMC5525906 28810548

[21] Bach JF . The hygiene hypothesis in autoimmunity: the role of pathogens and commensals. Nat Rev Immunol. 2018;18:105‐120. 29034905 10.1038/nri.2017.111

[22] Smallwood TB , Giacomin PR , Loukas A , Mulvenna JP , Clark RJ , Miles JJ . Helminth Immunomodulation in Autoimmune Disease. Front Immunol. 2017;8:453. 28484453 10.3389/fimmu.2017.00453 PMC5401880

[23] Anna M , Cheng DJ , Thomas S , Wilson JK , Parker W . Overcoming evolutionary mismatch by self‐treatment with helminths: current practices and experience. J Evol Med. 2015;3:1‐22.

[24] Santiago HC , Nutman TB . Human helminths and allergic disease: the hygiene hypothesis and beyond. Am J Trop Med Hyg. 2016;95:746‐753. 27573628 10.4269/ajtmh.16-0348 PMC5062766

[25] Shirvan SP , Borji H , Movassaghi A , et al. Anti‐inflammatory potentials of excretory/secretory (ES) and somatic products of Marshallagia marshalli on allergic airway inflammation in BALB/c mice. Iranian J Parasitol. 2016;11:515‐526. PMC5251180 28127363

[26] Kitagaki K , Businga TR , Racila D , Elliott DE , Weinstock JV , Kline JN . Intestinal helminths protect in a murine model of asthma. J Immunol. 2006;177:1628‐1635. 16849471 10.4049/jimmunol.177.3.1628

[27] Kim SE , Kim JH , Min BH , Bae YM , Hong ST , Choi MH . Crude extracts of Caenorhabditis elegans suppress airway inflammation in a murine model of allergic asthma. PLoS ONE. 2012;7:e35447. 22558152 10.1371/journal.pone.0035447 PMC3338843

[28] Schabussova I , Ul‐Haq O , Hoflehner E , et al. Oesophagostomum dentatum extract modulates T cell‐dependent immune responses to bystander antigens and prevents the development of allergy in mice. PLoS ONE. 2013;8:e67544. 23844022 10.1371/journal.pone.0067544 PMC3699627

[29] Ren J , Hu L , Yang J , et al. Novel T‐cell epitopes on Schistosoma japonicum SjP40 protein and their preventive effect on allergic asthma in mice. Eur J Immunol. 2016;46:1203‐1213. 26840774 10.1002/eji.201545775

[30] Logan J , Navarro S , Loukas A , Giacomin P . Helminth‐induced regulatory T cells and suppression of allergic responses. Curr Opin Immunol. 2018;54:1‐6. 29852470 10.1016/j.coi.2018.05.007

[31] Harnett W . Secretory products of helminth parasites as immunomodulators. Mol Biochem Parasitol. 2014;195:130‐136. 24704440 10.1016/j.molbiopara.2014.03.007

[32] Pineda MA , Lumb F , Harnett MM , Harnett W . ES‐62, a therapeutic anti‐inflammatory agent evolved by the filarial nematode Acanthocheilonema viteae. Mol Biochem Parasitol. 2014;194:1‐8. 24671112 10.1016/j.molbiopara.2014.03.003

[33] Crowe J , Lumb FE , Harnett MM , Harnett W . Parasite excretory‐secretory products and their effects on metabolic syndrome. Parasite Immunol. 2017;39:e12410. 10.1111/pim.12410 28066896

[34] Collison LW , Workman CJ , Kuo TT , et al. The inhibitory cytokine IL‐35 contributes to regulatory T‐cell function. Nature. 2007;450:566‐569. 18033300 10.1038/nature06306

[35] Liu JQ , Liu Z , Zhang X , et al. Increased Th17 and regulatory T cell responses in EBV‐induced gene 3‐deficient mice lead to marginally enhanced development of autoimmune encephalomyelitis. J Immunol. 2012;188:3099‐3106. 22387555 10.4049/jimmunol.1100106 PMC3311737

[36] Tong H , Miyazaki Y , Yamazaki M , et al. Exacerbation of delayed‐type hypersensitivity responses in EBV‐induced gene‐3 (EBI‐3)‐deficient mice. Immunol Lett. 2010;128:108‐115. 20064562 10.1016/j.imlet.2010.01.001

[37] Shamji MH , Layhadi JA , Achkova D , et al. Role of IL‐35 in sublingual allergen immunotherapy. J Allergy Clin Immunol. 2018;143(3):1131‐1142.e4. 30053528 10.1016/j.jaci.2018.06.041

[38] Chenery AL , Antignano F , Burrows K , Scheer S , Perona‐Wright G , Zaph C . Low‐dose intestinal trichuris muris infection alters the lung immune microenvironment and can suppress allergic airway inflammation. Infect Immun. 2016;84:491‐501. 26644379 10.1128/IAI.01240-15 PMC4730564

[39] Harnett MM , Harnett W . Can parasitic worms cure the modern world's ills? Trends Parasitol. 2017;33:694‐705. 28606411 10.1016/j.pt.2017.05.007

[40] Gurram RK , Zhu J . Orchestration between ILC2s and Th2 cells in shaping type 2 immune responses. Cell Mol Immunol. 2019;16:225‐235. 30792500 10.1038/s41423-019-0210-8 PMC6460501

[41] Akdis CA , Akdis M . Advances in allergen immunotherapy: aiming for complete tolerance to allergens. Sci Transl Med. 2015;7:280ps6. 10.1126/scitranslmed.aaa7390 25810310

[42] Akdis M , Akdis CA . Therapeutic manipulation of immune tolerance in allergic disease. Nat Rev Drug Discov. 2009;8:645‐660. 19644474 10.1038/nrd2653

[43] Bettini M , Vignali DA . Regulatory T cells and inhibitory cytokines in autoimmunity. Curr Opin Immunol. 2009;21:612‐618. 19854631 10.1016/j.coi.2009.09.011 PMC2787714

[44] Pandiyan P , Zhu J . Origin and functions of pro‐inflammatory cytokine producing Foxp3+ regulatory T cells. Cytokine. 2015;76:13‐24. 26165923 10.1016/j.cyto.2015.07.005 PMC4969074

[45] Oh SA , Liu M , Nixon BG , et al. Foxp3‐independent mechanism by which TGF‐beta controls peripheral T cell tolerance. Proc Natl Acad Sci USA. 2017;114:E7536‐E7544. 28827353 10.1073/pnas.1706356114 PMC5594672

[46] Wei X , Zhang J , Gu Q , et al. Reciprocal expression of IL‐35 and IL‐10 defines two distinct effector treg subsets that are required for maintenance of immune tolerance. Cell Rep. 2017;21:1853‐1869. 29141218 10.1016/j.celrep.2017.10.090

[47] Oh S , Hwang ES . The role of protein modifications of T‐bet in cytokine production and differentiation of T helper cells. J Immunol Res. 2014;2014:589672. 24901011 10.1155/2014/589672 PMC4036734

[48] Way SS , Wilson CB . Cutting edge: immunity and IFN‐gamma production during Listeria monocytogenes infection in the absence of T‐bet. J Immunol. 2004;173:5918‐5922. 15528324 10.4049/jimmunol.173.10.5918

[49] Maizels RM , McSorley HJ . Regulation of the host immune system by helminth parasites. J Allergy Clin Immunol. 2016;138:666‐675. 27476889 10.1016/j.jaci.2016.07.007 PMC5010150

