# Supplementary material for: Schistosoma japonicum peptide SJMHE1 suppresses airway inflammation of allergic asthma in mice
Source: J Cell Mol Med. 2019 Sep 9;23(11):7819–29. doi: 10.1111/jcmm.14661 (PMC6815837; doi:10.1111/jcmm.14661)
Supplement: Supplementary file 1 [file JCMM-23-7819-s001.pdf]

SUPPORTING INFORMATIONS

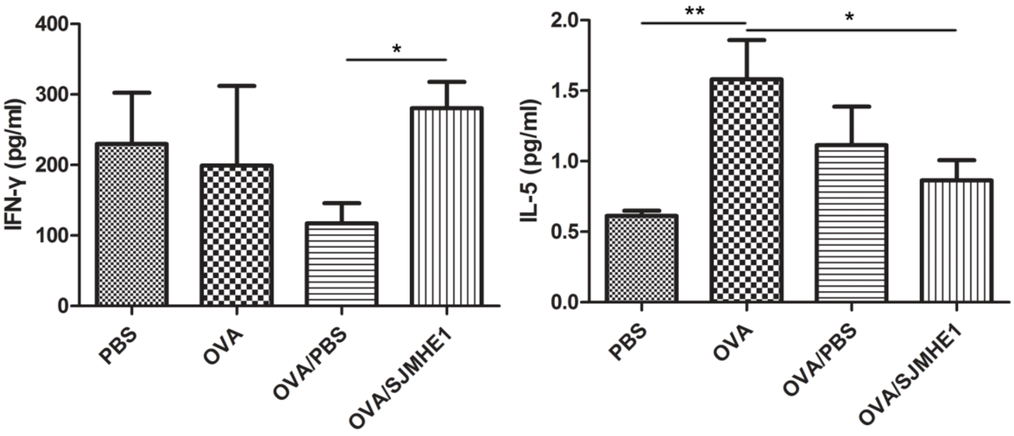

Figure S1 SJMHE1 treatment modulates IFN-γ and IL-5 production in the splenocytes of allergic mice. Splenocytes (2×10^5) of mice were stimulated by 50 μg/mL OVA for 48 h at 37 ℃. The supernatant was collected to detect IFN-γ and IL-5 by using the LEGENDplex™ flow cytometry bead array kit (BioLegend). Each result is the mean ± S.E.M. of two experiments performed in triplicate wells. Significance was analyzed by one-way ANOVA with Bonferroni test. **P* < 0.05, ***P* < 0.01.

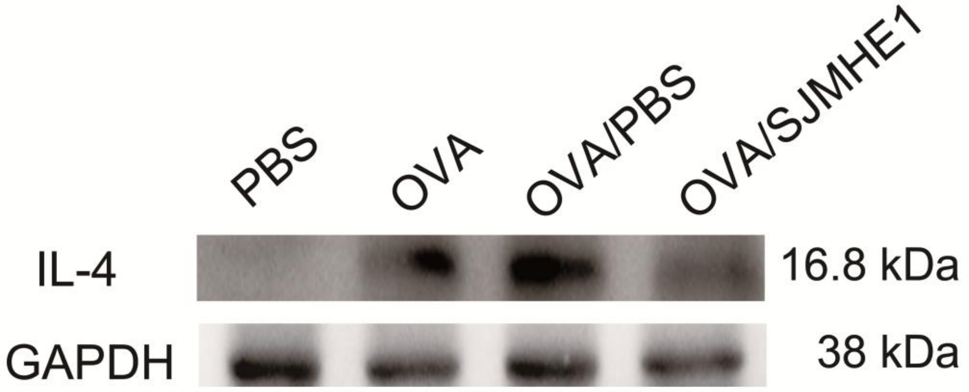

Figure S2 SJMHE1 treatment downregulates IL-4 expression in the lungs of allergic mice. The proteins of lung tissues in mice were extracted, and anti-IL-4 (1:250 dilution) (eBioscience, California, USA) and GAPDH (1:1000 dilution) (Cell Signaling Technology, Boston, USA) were used as the primary antibodies. IL-4 protein expression was quantified through Western blot analysis. A representative blot from four groups is shown.

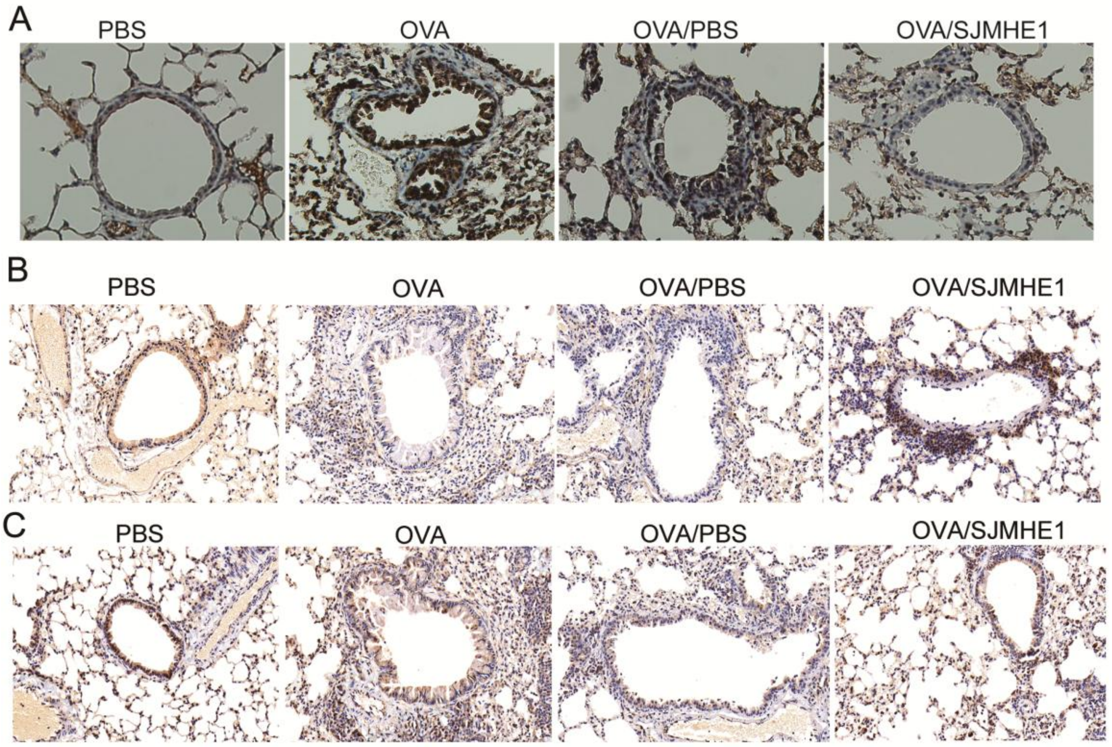

Figure S3 SJMHE1 treatment modulates IL-17, IL-10, and TGF-β expression in the lungs of allergic mice. The lungs of mice were stained with anti-IL-17 (1:600 dilution) (eBioscience, California, USA), anti-IL-10 (1:300 dilution) (Servicebio, Wuhan, China), and anti-TGF-β (1:500 dilution) (Servicebio, Wuhan, China). (A) IL-17, (B) IL-10, and (C) TGF-β-positive sites were stained brown with an immunohistochemical stain. Images are representatives of two independent experiments (*n* = 3 mice per group).

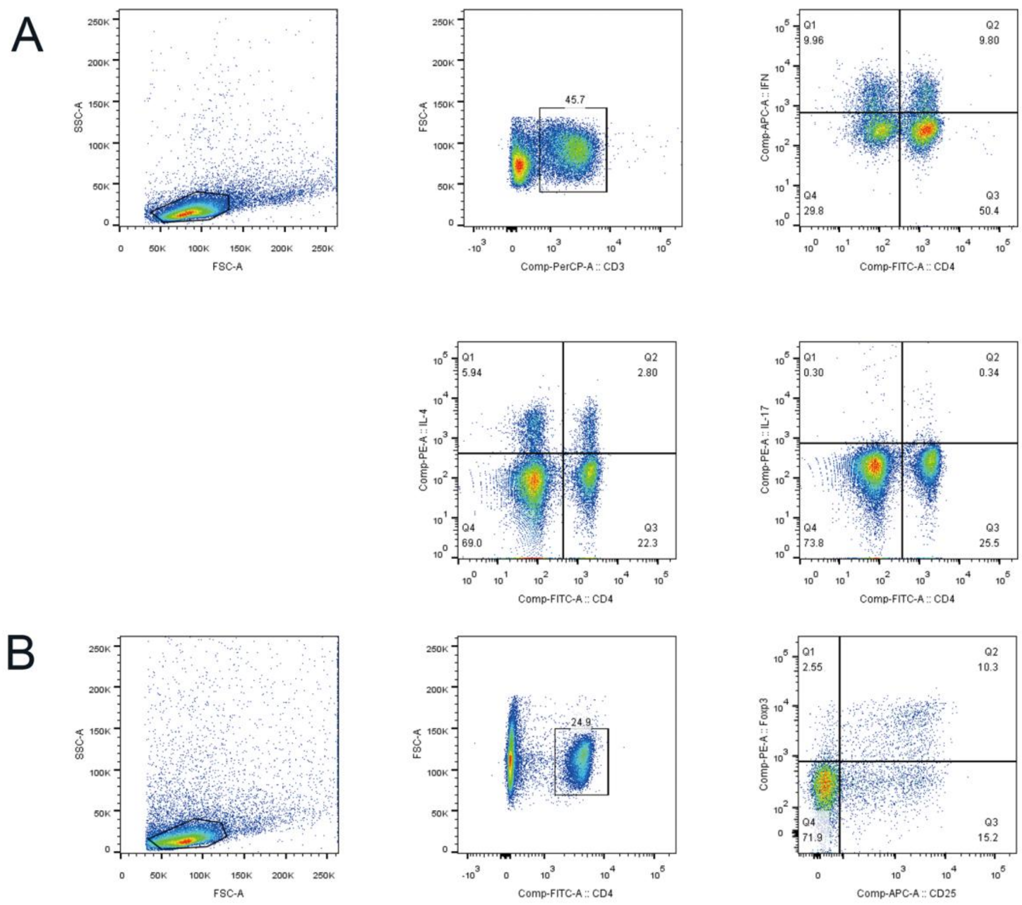

Figure S4 Gating strategies. Isolated cells were pre-gated on CD3$^+$ cells. (A) Gating strategy for analyzing CD4$^+$IFN$^+$ Th1, CD4$^+$IL-4$^+$ Th2, and CD4$^+$IL-17$^+$ Th17 cells in a representative sample of mice. (B) Gating strategy for analyzing CD4$^+$CD25$^+$Foxp3$^+$ Tregs in a representative sample of mice.
